# Tonsillar Microbiota: a Cross-Sectional Study of Patients with Chronic Tonsillitis or Tonsillar Hypertrophy

**DOI:** 10.1128/mSystems.01302-20

**Published:** 2021-03-09

**Authors:** Shengru Wu, Lalle Hammarstedt-Nordenvall, Mattias Jangard, Liqin Cheng, Sebastian Alexandru Radu, Pia Angelidou, Yinghua Zha, Marica Hamsten, Lars Engstrand, Juan Du, Anders Ternhag

**Affiliations:** a Centre for Translational Microbiome Research, Department of Microbiology, Tumor and Cell Biology, Karolinska Institutet, Stockholm, Sweden; b Department of Clinical Sciences, Intervention and Technology, Karolinska Institutet, Stockholm, Sweden; c Department of Ear, Nose and Throat (ENT), Karolinska University Hospital, Stockholm, Sweden; d ENT Unit, Research Laboratory, Sophiahemmet University, Stockholm, Sweden; e Department of Medicine, Solna, Karolinska Institutet, Stockholm, Sweden; f Department of Infectious Diseases, Karolinska University Hospital, Stockholm, Sweden; Cleveland Clinic

**Keywords:** chronic tonsillitis, tonsillar hypertrophy, tonsillar tissues, microbiota, 16S rRNA gene sequencing

## Abstract

Chronic tonsillitis (CT) and tonsillar hypertrophy (TH) are common tonsillar diseases that are related to infection and inflammation. Little is known about tonsillar microbiota and its role in CT and TH. This study aims to identify palatine tonsillar microbiota both on the surface and in the core tissues of CT and TH patients. In total, 22 palatine tonsils were removed and collected from CT and TH patients who underwent surgery. The surface and core microbiota in the tonsils of CT and TH patients were compared using 16S rRNA gene sequencing of V3-V4 regions. Differential tonsillar microbiotas were found in the CT versus TH patients and surface versus core tissues. Further, a higher relative abundance of bacterial genera, including *Haemophilus*, *Streptococcus*, *Neisseria*, *Capnocytophaga*, *Kingella*, *Moraxella*, and *Lachnospiraceae* [G-2] in patients with TH and *Dialister*, *Parvimonas*, *Bacteroidales* [G-2], *Aggregatibacter*, and *Atopobium* in patients with CT, was observed. Of these, the differential genera of *Dialister*, *Parvimonas*, and *Neisseria* served as key factors in the tonsillar microbiota network. Notably, four representable tonsillar microbial types were identified, with one, consisting of a higher abundance of *Haemophilus* and *Neisseria*, exclusively detected in the TH patients. This study analyzed the different tonsillar microbiota from the surface and core tissues of CT and TH patients. Several bacteria and various microbial types related to CT and TH were identified, along with potential bacterial networks and related immune pathways.

**IMPORTANCE** The human microbiota has been shown to be functionally connected to infectious and inflammation-related diseases. So far, only limited studies had been performed on tonsillar microbiota, although tonsils play an essential role in the human immune defense system and encountered numerous microorganisms. Our work presented different tonsillar microbiota from surface and core tissues of chronic tonsillitis (CT) and tonsillar hypertrophy (TH) patients. Notably, one tonsillar microbiota type, which contains a higher abundance of *Haemophilus* and *Neisseria*, was only detected in the TH patients. Furthermore, certain bacteria, such as *Haemophilus*, *Neisseria*, *Dialister*, and *Parvimonas*, may serve as microbial biomarkers to discriminate CT patients from TH patients. These data provide important microbiota data in the tonsillar research area and are highly useful for researchers both in the oral microbiome field and clinical field.

## INTRODUCTION

Palatine tonsils are important mucosa-associated and immunocompetent lymphoid organs located close to the respiratory and gastrointestinal tracts, which are teeming with microorganisms (1). The palatine tonsils could protect the body from the entry of exogenous material through mucosal sites. This condition also enables the passage of microorganisms through the epithelium, which attacks the immune system and causes tonsillar diseases (2). Chronic tonsillitis (CT) and tonsillar hypertrophy (TH), which lead to a sore throat or obstructive symptoms, including snoring and apnea, are the most common causes for tonsillectomy. CT is defined as a persistent and repetitive inflammation of the palatine tonsils and is characterized by infective symptoms (3). TH is more often accompanied by obstructive sleep apnea and no symptoms of infection (4). Although the clinical criteria for CT and TH have been published, the potential biomarkers to identify CT patients from TH patients and the pathogenesis behind the diseases are still under investigation (5).

So far, only a few microbiota studies have been performed on tonsils, especially tonsillar tissues, although they play an essential role in the human immune system and encounter numerous microorganisms all the time (1, 2, 6–8). Most of these studies were carried out using *in vitro* cultures of tonsillar bacteria (6, 7), but the culturing methods mostly ignored the less abundant or unculturable bacteria (6, 8). Other studies have used 16S rRNA gene sequencing with tonsillar swab samples, only considering surface microbiota (8–11). The composition of tonsillar microbiota may vary and play different roles in surface and core tonsillar tissues, and bacteria that cause tonsillitis may inhabit both the tonsillar surface and core tissue (6, 10). Identifying the differences between the tonsillar surface and the core tissue of the CT and TH patients provides detailed information on the potential development of the diseases from a microbiological perspective.

Thus, we aimed to identify the tonsillar microbiota both on the surface and in the core tissues of CT and TH patients using 16S rRNA gene sequencing of V3-V4 regions. Further comparison of the tonsillar microbiota of the four groups, namely, the core tonsil of chronic tonsillitis patients (C-CT), the surface tonsil of chronic tonsillitis patients (S-CT), the core tonsil of tonsillar hypertrophy patients (C-TH), and the surface tonsil of tonsillar hypertrophy patients (S-TH), was also carried out (see sample site in Fig. S1A in the supplemental material). This study provided a comprehensive view of tonsillar microbiota from both the core and surface tissues of CT and TH patients.

10.1128/mSystems.01302-20.1 FIG S1 Comparison of tonsillar (core tonsil and surface tonsil) microbial alpha diversity (ACE and Chao1 indices) in chronic tonsillitis (CT) and tonsillar hypertrophy (TH) patients. (A) Image of a lanced tonsil. The core and surface tissue areas are shown with yellow dotted lines. The sample site from core tissue is presented with a red dotted line, and the sample site from surface tissue is presented with a blue dotted line. (B) The microbial alpha diversity (ACE and Chao1 indices) in CT and TH patients was compared. The Mann-Whitney U test was carried out for comparing the two groups. (B) The microbial alpha diversity (ACE and Chao1 indices) in four groups, the core tonsil of chronic tonsillitis patients (C-CT), the surface tonsil of chronic tonsillitis patients (S-CT), the core tonsil of tonsillar hypertrophy patients (C-TH), and the surface tonsil of tonsillar hypertrophy patients (S-TH), was compared. Kruskal-Wallis test with Dunn’s *post hoc* tests was performed to compare all the groups. All the data are presented as mean ± standard deviation (SD) OTU counts. *, *p < *0.05. Download FIG S1, PDF file, 0.1 MB. Copyright © 2021 Wu et al. 2021 Wu et al. https://creativecommons.org/licenses/by/4.0/ This content is distributed under the terms of the Creative Commons Attribution 4.0 International license.

## RESULTS

### Differential microbiota diversity identified from the core and surface tonsils of CT and TH patients.

In total, 42 microbial DNA samples were successfully sequenced and used for further tonsillar microbiota analysis, including 10 samples of C-CT, 10 samples of S-CT, 10 samples of C-TH, and 12 samples of S-TH (Table 1). Two C-TH samples were excluded due to low library concentration.

**TABLE 1:** Clinical characteristics of the patients who underwent tonsillectomy due to CT or TH

Patient no.	Gender	Disease	Age (yr)	Source of sequenced DNA samples
1	Male	TH	2	Surface/core of left tonsil, surface of right tonsil
2	Male	TH	7	Surface/core of left tonsil, surface/core of right tonsil
3	Female	TH	5	Surface/core of left tonsil, surface of right tonsil
4	Missing	CT	10	Surface/core of left tonsil, surface/core of right tonsil
5	Male	TH	6	Surface/core of left tonsil, surface/core of right tonsil
6	Male	CT	30	Surface/core of left tonsil, surface/core of right tonsil
7	Male	TH	6	Surface/core of left tonsil, surface/core of right tonsil
8	Male	CT	9	Surface/core of left tonsil, surface/core of right tonsil
9	Male	CT	22	Surface/core of left tonsil, surface/core of right tonsil
10	Female	CT	29	Surface/core of left tonsil, surface/core of right tonsil
11	Female	TH	2	Surface/core of left tonsil, surface/core of right tonsil

**FIG 1:**
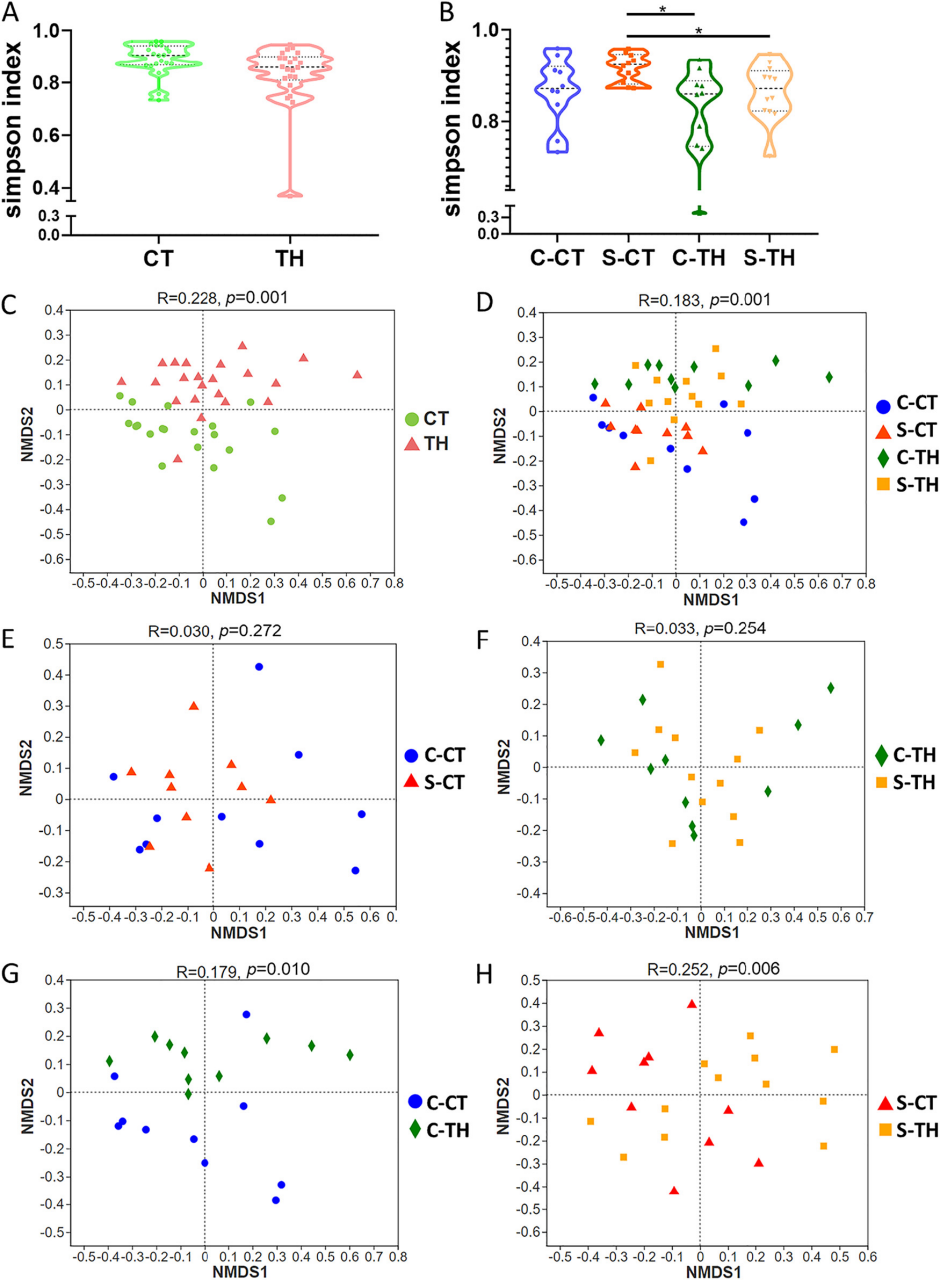
Comparison of tonsillar (core tonsil and surface tonsil) microbial alpha diversity (Simpson index) and beta diversity (nonmetric multidimensional scaling [NMDS] analysis with operational taxonomic units [OTU] based on Bray-Curtis distance matrix) between chronic tonsillitis (CT) and tonsillar hypertrophy (TH) patients. (A) The microbial alpha diversity was compared between the patient groups of CT and TH. The Mann-Whitney U test was carried out for comparing the two groups. (B) The microbial alpha diversity was compared between four groups, i.e., the core tonsil of chronic tonsillitis patients (C-CT), the surface tonsil of chronic tonsillitis patients (S-CT), the core tonsil of tonsillar hypertrophy patients (C-TH), and the surface tonsil of tonsillar hypertrophy patients (S-TH). Only S-CT was found to have significantly higher microbial alpha diversity than C-TH and S-TH. The Kruskal-Wallis test with Dunn’s *post hoc* test was performed to compare all the groups. (C) A significant difference in the microbial beta diversity was observed in the CT patients and TH patients. (D) Significant microbial beta diversity difference was seen in the C-CT, S-CT, C-TH, and S-TH groups. (E) The microbial beta diversity was compared between tissues collected from the core and surface tonsils of the CT patients, and no significant difference was observed. (F) The microbial beta diversity was compared between the tissues collected from the core and surface tonsils of TH patients, and no obvious difference was observed. (G) The microbial beta diversity was compared between the tissues collected from the core tonsils of the CT and TH patients, and a clear separation was seen. (H) The microbial beta diversity was significantly different between the tissues collected from the surface tonsils of the CT and TH patients. All the data in panels A and B are presented as means ± standard deviations (SD) from the OTU counts. *, *p < *0.05. For panels C to H, ANOSIM test was performed for comparing different groups.

First, microbial alpha diversity was tested using Simpson, abundance-based coverage estimator (ACE), and Chao1 indices. No significant differences in the microbial diversity were observed between CT and TH patients (Fig. 1A; see also Fig. S1B in the supplemental material). However, by further dividing the samples into four groups, C-CT, S-CT, C-TH, and S-TH, we found higher microbiota diversity (Simpson) in S-CT than C-TH and S-TH (Fig. 1B). Additionally, the Chao 1 and ACE indices showed higher microbial community richness in S-CT, C-TH, and S-TH than C-CT (Fig. S1C). Overall, the microbiota richness and diversity of each sample, although slightly different, with some reaching significant levels, were very similar, indicating a comparable alpha diversity of microbiome in tonsils.

The comparison of microbiota beta diversity between CT and TH patients through nonmetric multidimensional scaling (NMDS) analysis based on the identified operational taxonomic units (OTUs) presented a significant dissimilarity (p_ANOSIM_ [analysis of similarity] = 0.001) (Fig. 1C). Further, a significant level of separation (p_ANOSIM_ = 0.001) was also observed when the patients were assigned to the C-CT, S-CT, C-TH, and S-TH groups (Fig. 1D). The microbiota from the core and surface tonsillar tissues of the same disease type were found to be similar (Fig. 1E and F). Notably, both the core and surface tonsillar microbiota could be used to distinguish the CT patients from the TH patients (p_ANOSIM_ = 0.010 and 0.006) (Fig. 1G and H). Furthermore, although age and gender influence microbiota diversity, by excluding the potential interferences from age and gender, significant dissimilarity of microbiota was still observed (Table S1). Overall, the tonsillar microbial communities significantly differ in the CT and TH patient groups.

10.1128/mSystems.01302-20.4 TABLE S1 Different microbial composition of core or surface tonsils from chronic tonsillitis (CT) or tonsillar hypertrophy (TH) patients based on a permutational multivariate analysis of variance (PERMANOVA). Download Table S1, DOCX file, 0.02 MB. Copyright © 2021 Wu et al. 2021 Wu et al. https://creativecommons.org/licenses/by/4.0/ This content is distributed under the terms of the Creative Commons Attribution 4.0 International license.

### Genera and species composition identified from the surface and core tonsils of CT and TH patients.

**FIG 2:**
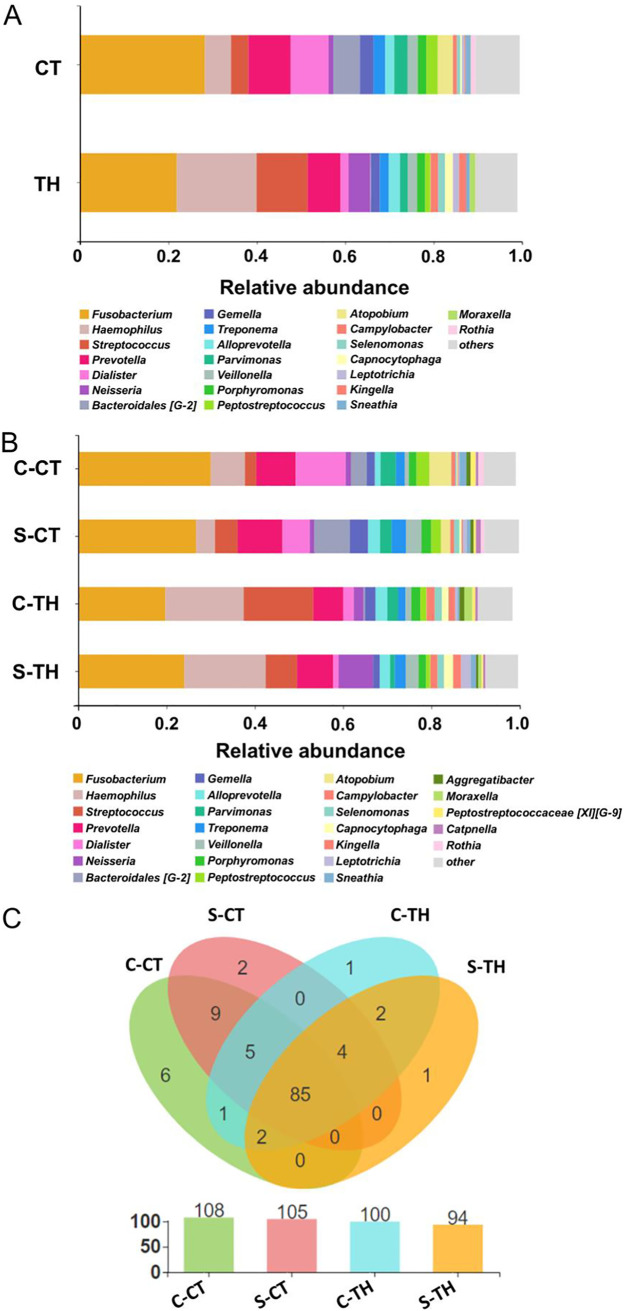
Microbial composition at the genus level in CT and TH patients. (A) The microbial genus composition was compared between the patient groups of CT and TH. (B) The microbial genus composition was compared between the C-CT, S-CT, C-TH, and S-TH groups. (C) The unique and common genera between the C-CT, S-CT, C-TH, and S-TH groups. Total genus numbers detected in each group are listed below. Only genera with relative abundance of more than 1% are listed in panels A and B.

A total of 118 genera and 256 species were identified in all the tonsillar samples (Fig. 2 and Fig. S2). The top four main genera that were found in the tonsil samples were *Fusobacterium*, *Haemophilus*, *Streptococcus*, and *Prevotella* (Fig. 2A and B). Of the 118 genera, 17 and 4 genera were identified only in CT and TH patients, respectively (Fig. 2C). Ten genera were identified in only one of the four groups (C-CT, S-CT, C-TH, and S-TH) (Fig. 2C). Among the 256 species, Fusobacterium nucleatum subsp. *vincentii*, Haemophilus influenzae, Dialister invisus, and Parvimonas micra were the top defined species that were detected in tonsillar tissues from both CT and TH patients (Fig. S2). Moreover, the *Bacteroidales* [G-2] bacterium HMT 274 and Streptococcus pyogenes were identified as the most abundant species that were detected only in the CT and TH patients, respectively (Fig. S2).

10.1128/mSystems.01302-20.2 FIG S2 Comparison of the tonsillar (core tonsil and surface tonsil) microbial communities at the species level in CT and TH patients. (A) The microbial species in the CT and TH patients were compared. (B) The microbial species in the C-CT, S-CT, C-TH, and S-TH groups were compared. Only species abundance of more than 1% are listed in panels A and B. Download FIG S2, TIF file, 2.8 MB. Copyright © 2021 Wu et al. 2021 Wu et al. https://creativecommons.org/licenses/by/4.0/ This content is distributed under the terms of the Creative Commons Attribution 4.0 International license.

### Four tonsillar microbial types identified.

**FIG 3:**
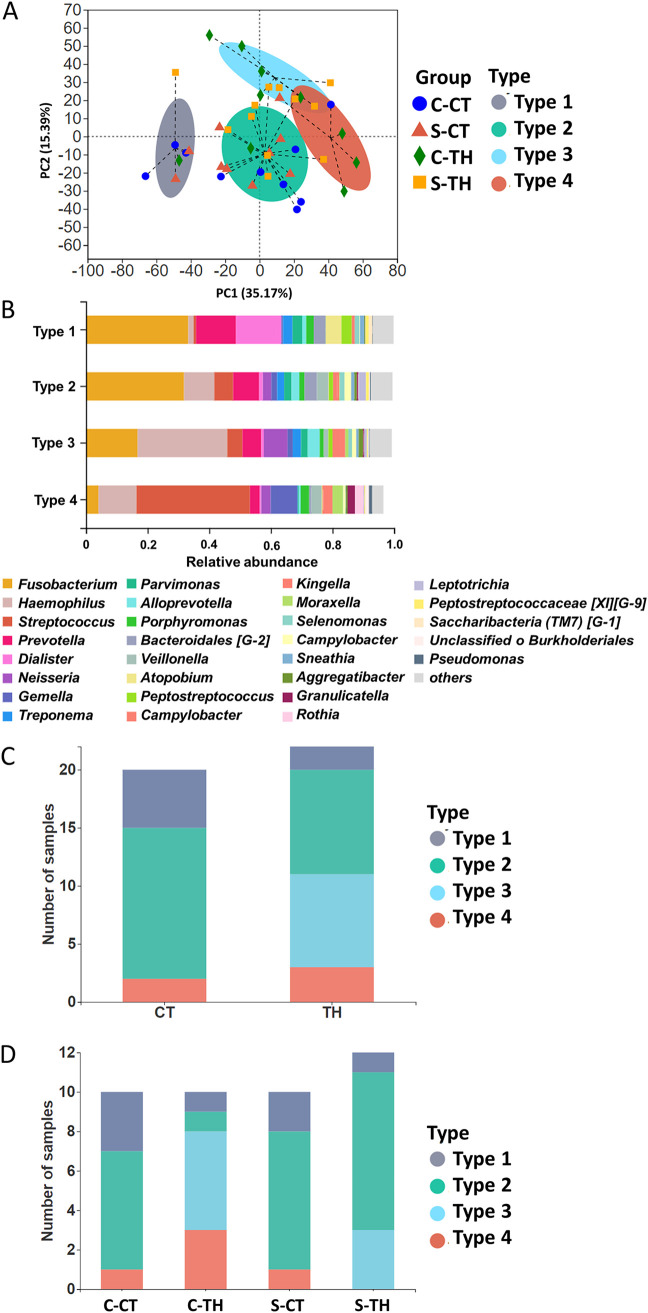
Tonsillar microbial type distribution in tonsillar (core tonsil and surface tonsil) tissues from CT and TH patients using partitioning around medoids (PAM) clusters based on the Jensen-Shannon distance (JSD) and Calinski-Harabasz (CH) indices. (A) Four different tonsillar microbial types were identified based on the genera of tonsillar microbes from CT and TH patients. (B) The microbial genus composition was compared between four different tonsillar microbial types. Only genera with relative abundance of more than 1% are listed. (C) The microbial types distribution was compared between the CT and TH patients. (D) The microbial type distribution was compared between the C-CT, S-CT, C-TH, and S-TH groups.

According to the similarities in the genera identified from different samples, four different tonsillar microbial types were identified (Fig. 3A): type 1 has higher abundance of *Fusobacterium*, *Dialister*, and *Prevotella*, type 3 has higher abundance of *Haemophilus* and *Neisseria*, type 4 has higher abundance of *Streptococcus* and *Gemella*, and type 2 has an abundance similar to those of the abovementioned genera (Fig. 3B). Notably, type 3 was identified only in the TH patients (Fig. 3C). Further, type 4 was not detected in the S-TH group (Fig. 3D). These results were mainly due to the CT group not containing as abundant *Haemophilus* and *Neisseria* as the TH patients (Fig. 2A and 3A and B).

### Certain bacteria, especially from the surface tonsils, help distinguish CT patients from TH patients.

**FIG 4:**
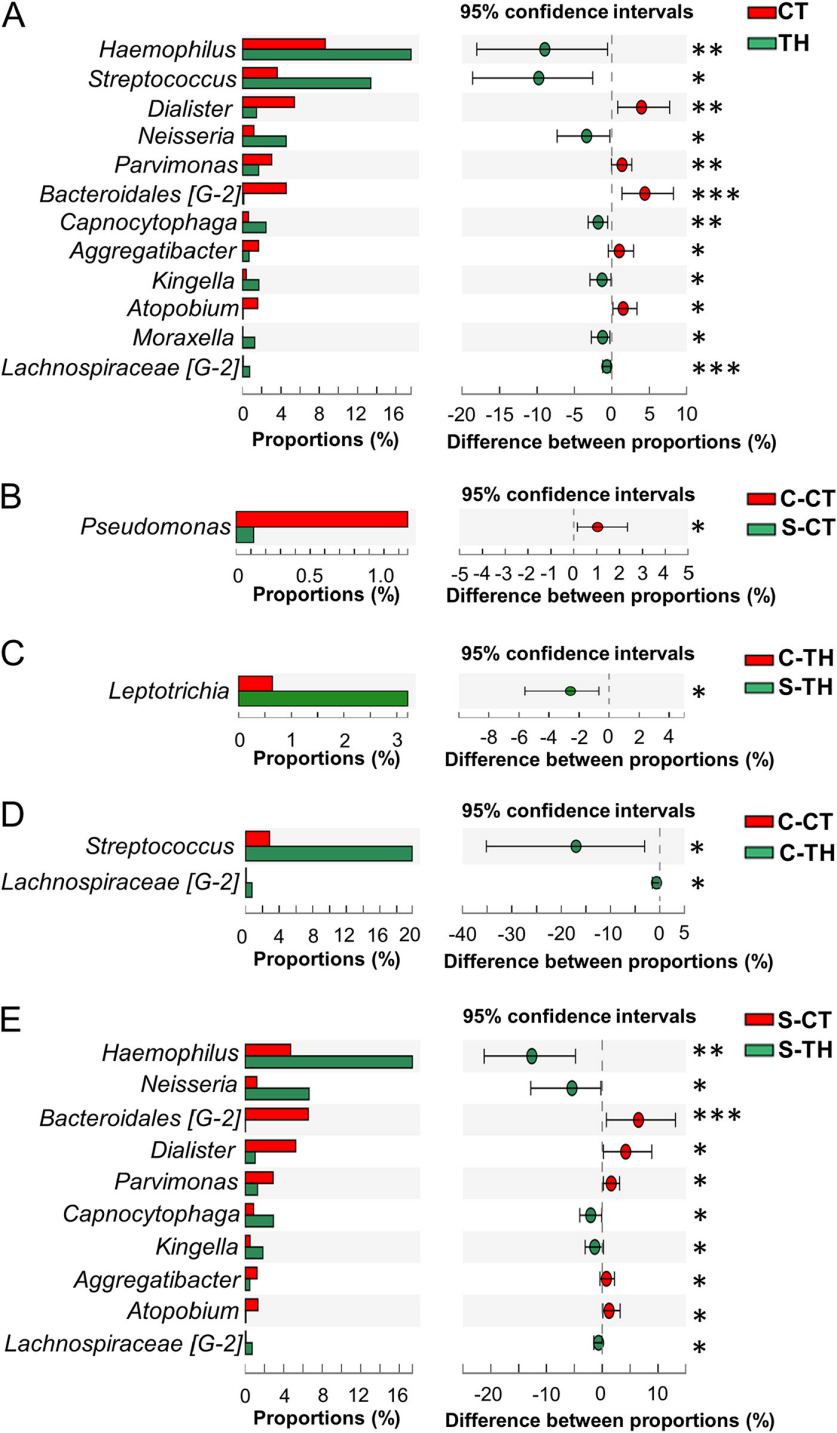
Differential genera identified in the comparison between tonsillar (core tonsil and surface tonsil) tissues from the CT and TH patients. (A) Differential genera selected from the comparison between patient groups of CT and TH patients. (B) Differential genera selected from the comparison between the tissues collected from the core and surface tonsils of the CT patients. (C) Differential genera selected from the comparison between the tissues collected from the core and surface tonsils of the TH patients. (D) Differential genera selected from the comparison between the tissues collected from the core tonsils of the CT and TH patients. (E) Differential genera selected from the comparison between the tissues collected from the surface tonsils of the CT and TH patients. Differential genera with a relative abundance of more than 1% and false discovery rate (FDR) of <0.05 were selected using multiple comparisons of the Mann-Whitney U test with a Benjamini-Hochberg adjustment. *, FDR < 0.05; **, FDR < 0.01; ***, FDR < 0.001.

As many as 12 different genera were identified as significantly different in their proportions when comparing the tonsillar tissues of the CT and TH patients (Fig. 4A). Of these, *Dialister*, *Parvimonas*, *Bacteroidales* [G-2], *Aggregatibacter*, and *Atopobium*, from the tonsillar tissues of the CT patients, and *Haemophilus*, *Streptococcus*, *Neisseria*, *Capnocytophaga*, *Kingella*, *Moraxella*, and *Lachnospiraceae* [G-2], from the tonsillar tissues of the TH patients, were significantly higher than those of the other group of patients (Fig. 4A).

Further analysis of the groups (C-CT, S-CT, C-TH, and S-TH) showed that most of the differences between CT and TH patients were also observed by comparing only the surface microbiota (Fig. 4B to E). A significantly larger amount of *Streptococcus* was found in the core tissues of the TH patients than in the core tissues of the CT patients (Fig. 4D). Furthermore, as shown in Fig. 4E, when comparing the S-CT with the S-TH group, a higher level of *Bacteroidales* [G-2], *Dialister*, *Parvimonas*, *Aggregatibacter*, and *Atopobium* from the surface tonsillar tissues of the CT patients and a higher level of *Haemophilus*, *Neisseria*, *Capnocytophaga*, *Kingella*, and *Lachnospiraceae* [G-2] from the surface tonsillar tissues of the TH patients were observed. Comparisons among other groups did not reveal substantial microbial differences (Fig. 4B and C).

At the species level, significantly higher levels of Parvimonas micra, *Bacteroidales* [G-2] bacterium HMT 274, Dialister invisus, Atopobium rimae, and Catonella morbi in the CT patients and Haemophilus influenzae, Streptococcus pyogenes, Neisseria flavescens, *Kingella* sp. strain HMT 012, Campylobacter rectus, Capnocytophaga sputigena, *Lachnospiraceae* [G-2] bacterium HMT 088, and Prevotella nanceiensis in the TH patients were detected (Fig. S3A). Similar to what was observed at the genus level, most of the differences could be observed from the surface tissues of the CT and TH patients, while the other comparisons had much less bacterial difference (Fig. S3B to E).

10.1128/mSystems.01302-20.3 FIG S3 Identified differential species when the tonsillar (core tonsil and surface tonsil) tissues from CT and TH patients were compared. (A) Differential species selected from the comparison between CT and TH patients. (B) Differential species selected from the comparison between tissues collected from the core and surface tonsils of the CT patients. (C) Differential species selected from the comparison between the tissues collected from the core and surface tonsils of the TH patients. (D) Differential species selected from the comparison between the tissues collected from the core tonsils of the CT and TH patients. (E) Differential species selected from the comparison between the tissues collected from the surface tonsils of the CT and TH patients. Differential genera with a relative abundance of more than 1% and false discovery rate of <0.05 were selected using multiple comparisons of the Mann-Whitney U test with a Benjamini-Hochberg adjustment. *, FDR < 0.05; **, FDR < 0.01; ***, FDR < 0.001. Download FIG S3, PDF file, 2.9 MB. Copyright © 2021 Wu et al. 2021 Wu et al. https://creativecommons.org/licenses/by/4.0/ This content is distributed under the terms of the Creative Commons Attribution 4.0 International license.

### The bacteria differed between CT and TH patients and served various functions.

**FIG 5:**
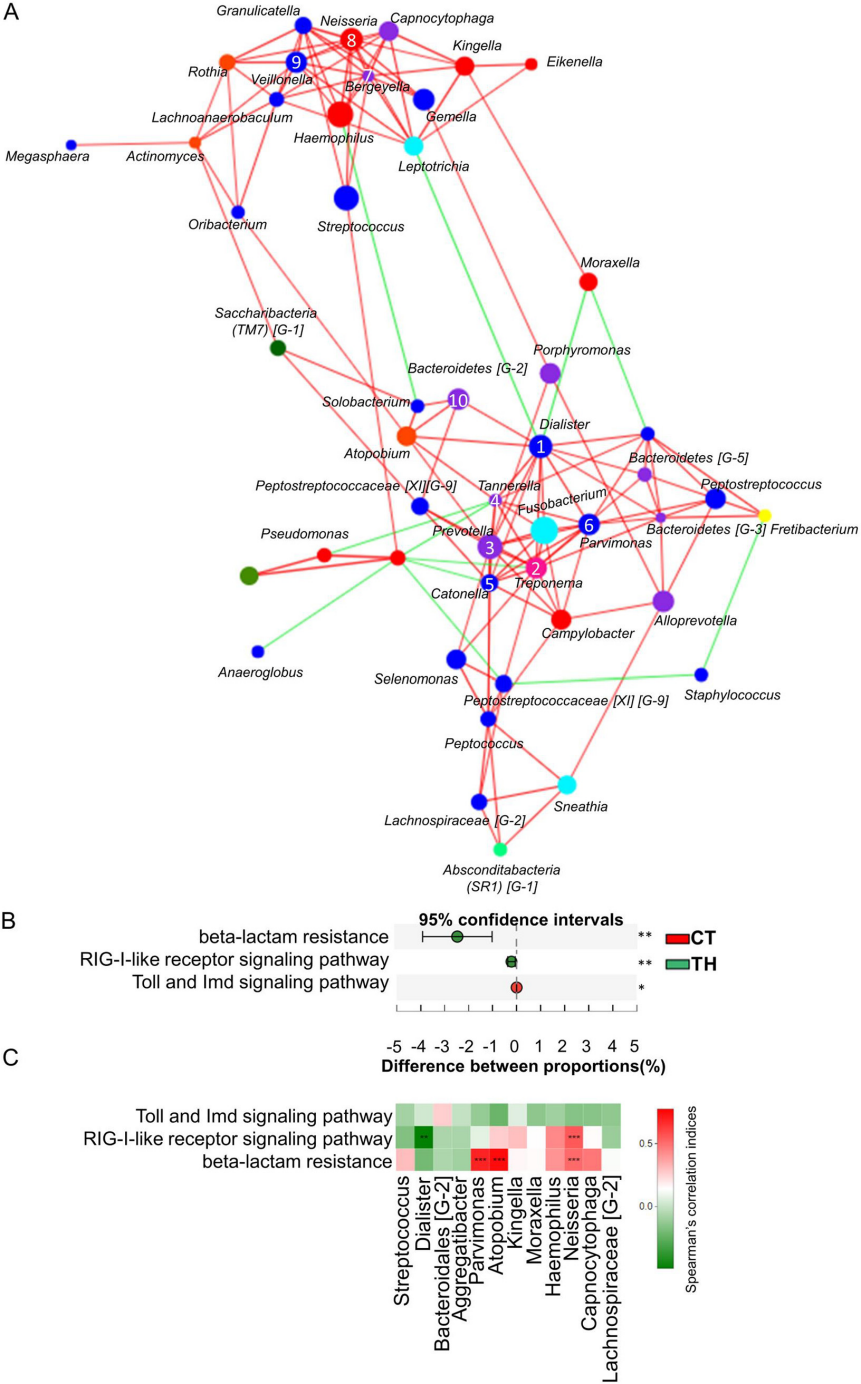
Identification of the tonsillar microbial network and potential drug resistance and immune function-related pathways. (A) The correlation among genera in tonsillar tissues based on Spearman's rank correlation coefficient analysis (correlation coefficient >0.5 and *p < *0.05). The numbers on the nodes represent the rank of top 10 key genera based on the calculated degree centrality, closeness centrality, and betweenness centrality. The red line represents the positive correlation, and the green line represents the negative correlation. (B) The identified different drug resistance- and immune function-related pathways based on the predicted metagenome comparison of tonsillar tissues from CT and TH patients. *, FDR < 0.05; **, FDR < 0.01. (C) The correlation between significantly altered pathways and genera. Only Spearman's rank correlation coefficient of >0.5 and FDR of <0.05 are presented. The red grid indicates the positive correlation, and the green grid indicates the negative correlation. **, FDR < 0.01; ***, FDR < 0.001.

From the network generated based on all the genera and the degree centrality, closeness centrality, and betweenness centrality of each genus, bacteria that had significantly different proportions between CT and TH patients, such as *Dialister*, *Parvimonas*, and *Neisseria*, were located at key positions in the microbiota network (Fig. 5A, Table S2). This indicates that, apart from distinguishing CT patients from HT ones, the identified bacteria also serve core functions in the microbiota network. Furthermore, Fig. 5A shows that the majority of the correlation was positively related, indicating that the abundance alteration of these key genera positively affected the other bacteria in the tonsillar microbiota network.

10.1128/mSystems.01302-20.5 TABLE S2 Identification of key genera based on calculated degree centrality, closeness centrality, and betweenness centrality using Python package “NetworkX”. Download Table S2, DOCX file, 0.01 MB. Copyright © 2021 Wu et al. 2021 Wu et al. https://creativecommons.org/licenses/by/4.0/ This content is distributed under the terms of the Creative Commons Attribution 4.0 International license.

Different drug resistance (antimicrobial and antineoplastic) and immune pathways were further analyzed. Significantly higher beta-lactam resistance and increased retinoic acid-inducible gene I (RIG-I)-like receptor signaling pathways were observed in the TH patients, while an increase of Toll/Imd signaling pathways was observed in the CT patients (Fig. 5B). Furthermore, *Neisseria*, which was significantly increased in TH patients, was also correlated with an increased RIG-I-like receptor signaling pathway and beta-lactam resistance in the TH patients. *Parvimonas* and *Atopobium*, which were significantly increased in CT patients, were correlated with a higher beta-lactam resistance (Fig. 5C). In addition, *Dialister*, which was increased in the CT patients, was negatively correlated with the RIG-I-like receptor signaling pathway (Fig. 5C).

## DISCUSSION

In this study, we have demonstrated the microbial differences between surface and core tonsillar tissue and their relation to CT and TH diseases using microbiome 16S rRNA gene sequencing. Our data indicated a comparable bacterial alpha diversity of tonsillar microbiota from CT and TH patients. However, the microbial communities present in the tonsillar tissues of CT patients are different from those in TH patients, especially the ones on the surface of the tonsils. We proposed four different tonsillar microbial types and certain bacterial genera/species that could distinguish CT patients from TH patients. Furthermore, the key players in the microbiota and bacterial functions have also been investigated.

Our study used surgically removed tonsillar samples, which provided more material than tonsillar swabs and allowed us to compare surface tissues with core tissues. The culture-independent sequencing techniques could also draw a complete picture of microbiota in tonsils, especially for the unculturable bacteria. Moreover, by using the expanded Human Oral Microbiome Database (eHOMD) to identify the tonsillar genera, we found a relatively high abundance of *Haemophilus*, *Streptococcus*, *Neisseria*, *Capnocytophaga*, *Kingella*, *Moraxella*, and *Lachnospiraceae* [G-2] in patients with TH and *Dialister*, *Parvimonas*, *Bacteroidales* [G-2], *Aggregatibacter*, and *Atopobium* in patients with CT (Fig. 4). These identified genera are consistent with earlier studies on tonsillar microbiota. One study with recurrent tonsillitis (RT) participants showed a high relative abundance of *Parvimonas*, while patients with obstructive sleep apnea (OSA) had a high relative abundance of *Haemophilus* and *Capnocytophaga* (8). Other studies have also identified that patients with OSA or TH have a higher relative abundance of *Haemophilus* (7) and *Streptococcus* (12) than RT patients. Moreover, the main species of *Moraxella* (13) and *Neisseria* (9) have also proven to be positively correlated with the occurrence of TH in previous research. *Dialister* (14), *Aggregatibacter* (15), and *Atopobium* (16), which were present significantly more in CT patients, have been demonstrated to be increased in several infective and inflammation-related diseases, including acute tonsillitis (16). Notably, *Haemophilus* and *Neisseria*, which have higher abundance in tonsillar microbiota type 3, were significantly increased in the TH patients, highlighting the potential role of these bacteria in the occurrence of TH (8, 9).

Of the significantly different species among the compared groups, Haemophilus influenzae and Streptococcus pyogenes have been more frequently observed among TH patients than CT patients (6). Additionally, *Bacteroidales* [G-2] bacterium HMT 274 (17), Campylobacter rectus (18), Capnocytophaga sputigena (19), Atopobium rimae (20), and Dialister invisus (21) were previously identified as commensals involved in oral infections. All these results indicate that the presented bacteria serve as biomarkers for the identification of TH and CT.

Antimicrobial resistance has become a concern and threat to health benefits, reflecting the worldwide abuse of antibiotics. Whether the resistance predicted in this study is related to intrinsic resistance or antibiotic treatment should be further considered (22). Furthermore, several bacteria found in this study have been shown to correlate with pathogenicity and inflammation in tonsillitis (23). The immune response to acute tonsillitis-related microbes has also been reported (24). Toll-like receptors (TLRs) and RIG-I-like receptors (RLRs) are the key innate immune pattern recognition receptors and are regulated by elaborate mechanisms to ensure a beneficial outcome in response to pathogens (25). Significantly increased RLR signaling pathways and TLR pathways were observed in the TH and CT patients, respectively. This indicates that the differential bacterial composition of TH and CT patients activate different immune signaling pathways. Notably, bacteria such as *Neisseria*, which was enriched in the TH patients and triggers the RIG-I-like receptor signaling pathway, may serve as the key pathogenesis player for TH development. Further studies, especially longitudinal studies, are needed to illuminate the role and mechanism of the differential species in regulating the occurrence of different diseases.

There are several limitations to the present study. First, although significant differences between the patient groups were identified, this is a small cohort of patients, which must be taken into account when interpreting the data. Additionally, exposure to antibiotics affects microbiota, at least in the short or medium term. The patients’ history of antibiotic use was unknown, which also indicates that our microbiota findings might be influenced by different drugs administered before tonsillectomy. Furthermore, taxonomic classification is not very reliable at the species level, amplifying part of the 16S rRNA gene (V3-V4 regions) (26), and the use of phylogenetic investigation of communities by the reconstruction of unobserved states 2 (PICRUSt2) could not reflect the actual metagenome and microbial function changes. However, using PICRUSt2 could provide new microbial insights to search for potential biomarkers or treatment targets for CT or TH patients (27). Moreover, given that most of the identified differential genera in our study were also previously identified between CT and TH patients using swab samples (7–9), we believe that our results provide valuable information for further studies to use, especially the bacteria we found to be related to CT versus TH and core versus surface tissues. Longitudinal studies that are focused on the metagenome and that use information on factors that influence microbiota, such as antibiotic exposure and eating habits, will provide further understanding on tonsillar microbiota.

### Conclusions.

To summarize, we have demonstrated that significant levels of differential microbiota are present in the core and surface tonsillar tissues of CT and TH patients. Further, our results indicated that several identified genera, such as *Haemophilus*, *Neisseria*, *Dialister*, and *Parvimonas*, could be used as potential biomarkers and have a role in the pathogenesis of diseases.

## MATERIALS AND METHODS

### Study design, population, and sample collection.

This was a cross-sectional study involving patients who were operated upon for tonsillectomy between May and October 2015 at four ear, nose, and throat (ENT) clinics in Stockholm, Sweden. The CT and TH patients were diagnosed according to the Paradise’s (28) and Brodsky’s criteria (29). Patients with other otolaryngologic problems or systemic disorders were excluded. A total of 22 tonsils (both left and right sides) were removed and collected from 5 CT and 6 TH patients and directly snap-frozen at −80°C until further DNA extraction. Patients who had symptoms of recurrent acute tonsillitis (defined as multiple episodes of tonsillitis for more than 2 years), foul-smelling mouth, and recurrent sore throat were considered CT. The patients who had obstructive symptoms, such as breathing difficulties, swallowing problems, snoring, and enlarged tonsils, were considered TH. There were no tonsillar infection problems and no history of recurrent tonsillitis in the TH group. Detailed information about these patients is listed in Table 1. Participation in the study was voluntary, and informed consent was obtained before surgery. The study was approved by the Regional Ethical Board at Karolinska Institutet, Stockholm, Sweden.

### DNA extraction.

Both the surface and core tissues (around 0.5 cm by 0.5 cm in size) from each tonsil were cut and collected in a sterile environment. As shown in Fig. S1A in the supplemental material, there is a distinct boundary between the surface and core tissues of tonsils. The surface tissue of each tonsil was gathered, and then the tonsillar samples were prudently and sagitally sectioned into two parts, followed by the collection of the core tissues. Scalpels were changed between collection of surface and core tissues and between samples in a biosafety cabinet.

Tonsillar tissues (*n* = 44) were bead-beaten with Matrix E beads (MP Biomedicals, USA) for three 2-min cycles. The tissues were then incubated with 20 μl of lysozyme (100 mg/ml) at 37°C for 60 min, followed by digestion with 20 μl proteinase K (20 mg/ml) for 90 min at 55°C, 250 rpm. The purification was performed with TECAN Fredom EVO with the Quick DNA Magbead Plus kit (D4082; Zymo Research, USA), according to the manufacturer’s guidelines and our previous study (30, 31). During the DNA extraction process, DNA/RNA Shield and ZymoBIOMICS Microbial Community Bacterial Standard (D6300; Zymo Research, USA) were used as the negative and positive extraction controls, respectively. The final DNA concentration and purification were determined by fluorometry using a Qubit 2.0 fluorometer (Life Technologies, NY, USA). The purified DNA was stored at −80°C until further microbiota sequencing.

### Microbiota sequencing and analysis.

The V3-V4 regions of the 16S rRNA genes were amplified with Illumina sequencing index-binding primer pair 341F/805R using a previously established automated pipeline (31). For library preparation, DNA-free water and ZymoBIOMICS Microbial Community DNA Standard (D6305; Zymo Research, USA) were included as the negative and positive PCR controls. Meanwhile, both negative and positive controls from the extraction phase were also submitted to PCR as the negative and positive controls. Two (the core tissues of right tonsils of patients 1 and 3) of 44 sample libraries with concentrations less than 2 nM were discarded (Table 1). Thereafter, paired-end sequences (2 × 300 bp) of 42 prepared sample libraries and controls from the DNA extraction and PCR steps were generated on an Illumina MiSeq sequencing platform (Illumina, CA, USA) with MiSeq reagent kit v3 (Illumina).

### Bioinformatics analysis.

A power analysis of the sample size for 16S rRNA gene sequencing was performed using a microbiome power calculator (32). With a false discovery rate (FDR) of 5% for multiple testing corrections, the simulation results demonstrated that the 42 sequencing samples had a sufficient power of 0.8 to detect significant differences in community diversity.

Raw fastq files were quality filtered and then merged together using Trimmomatic (33) and FLASH (34). Briefly, the reads were deleted if they were less than 50 bp in length or received an average quality score of <20 over a 50-bp sliding window. Sequences were merged if the overlap was longer than 10 bp and the mismatch less than 2 bp. Reads were further removed when containing ambiguous bases.

A greedy algorithm using UPARSE (version 7.1; http://drive5.com/uparse/) that performs chimera filtering and OTU clustering simultaneously was applied (35). OTUs were clustered with 97% similarity. The OTUs with abundance lower than the negative controls were excluded for further analysis. The taxonomy of each OTU was analyzed by an RDP Classifier algorithm (http://rdp.cme.msu.edu/) against the eHOMD (V15.2) database (36) with a confidence threshold of 0.7. Different taxonomies were determined at the phylum, class, order, family, genus, and species levels for all the sequenced samples.

The analysis on alpha and beta diversity was performed on the filtered data using USEARCH alpha_div (37) and UniFrac metrics (38) in QIIME (version 1.9.1) (39), respectively. In detail, species OTU richness estimates (Chao1 and ACE) and diversity index (Simpson) were carried out for microbiota alpha diversity from all the samples. Beta diversity from different samples was compared via nonmetric multidimensional scaling (NMDS) analysis based on Bray-Curtis distance matrices. In addition, a permutational multivariate analysis of variance (PERMANOVA) with treatments (surface and core tonsils of CT and TH patients) and three covariates (age, gender, and heterogeneity of left/right tonsil tissues) were performed to compare the contribution of treatments and each covariate to the microbiota difference. Furthermore, partitioning around medoids (PAM) clustering was performed based on the Jensen-Shannon divergence (JSD). The best clustering K number was calculated using the Calinski-Harabasz (CH) index (40). The tonsillar microbial types were analyzed using between-class analysis (BCA) (K ≥ 3) and visualized by principal coordinate analysis (PCoA) (K ≥ 2).

Network graphs were calculated based on the correlation of the abundance of all the tested genera using the Python package NetworkX and visualized in Cytoscape (v3.4.0) (41, 42). Degree centrality, closeness centrality, and betweenness centrality were calculated and ranked (41, 43). The phylogenetic investigation of communities by the reconstruction of unobserved states 2 (PICRUSt2) analysis (https://github.com/picrust/picrust2) (27) was used to predict the metagenome in the samples and predict the bacterial drug resistance- and immune function-related pathways using the Kyoto Encyclopedia of Genes and Genomes (KEGG) orthologs (KEGG hierarchical level 3) (44).

### Statistics.

The Mann-Whitney U test was performed to compare the microbial alpha diversity of CT and TH. The Kruskal-Wallis test with Dunn’s *post hoc* test was employed to test microbial alpha diversity differences between C-CT, S-CT, C-TH, and S-TH. ANOSIM analysis based on Bray-Curtis distance matrices was used to identify the beta diversity between two or more compared groups. In addition, the Adonis function from the R package vegan was used for PERMANOVA (45, 46). The Mann-Whitney U test was used with multiple comparisons adjusted by the Benjamini-Hochberg FDR to rank bacteria that were significantly different in their genus/species levels and for drug resistance pathway analysis. Pairwise correlations (*p < *0.05; Spearman's rank correlation coefficient >0.5) were used to generate genus-level cooccurrence networks.

### Data availability.

All the data generated or analyzed for this study are included in this paper. The sequencing reads are available in the Sequence Read Archive (SRA) of NCBI under accession project number PRJNA633894.
