# Supplementary figures and images for: Tonsillar Microbiota: a Cross-Sectional Study of Patients with Chronic Tonsillitis or Tonsillar Hypertrophy
Source: mSystems. 2021 Mar 9;6(2):e01302-20. doi: 10.1128/mSystems.01302-20 (PMC8547005; doi:10.1128/mSystems.01302-20)

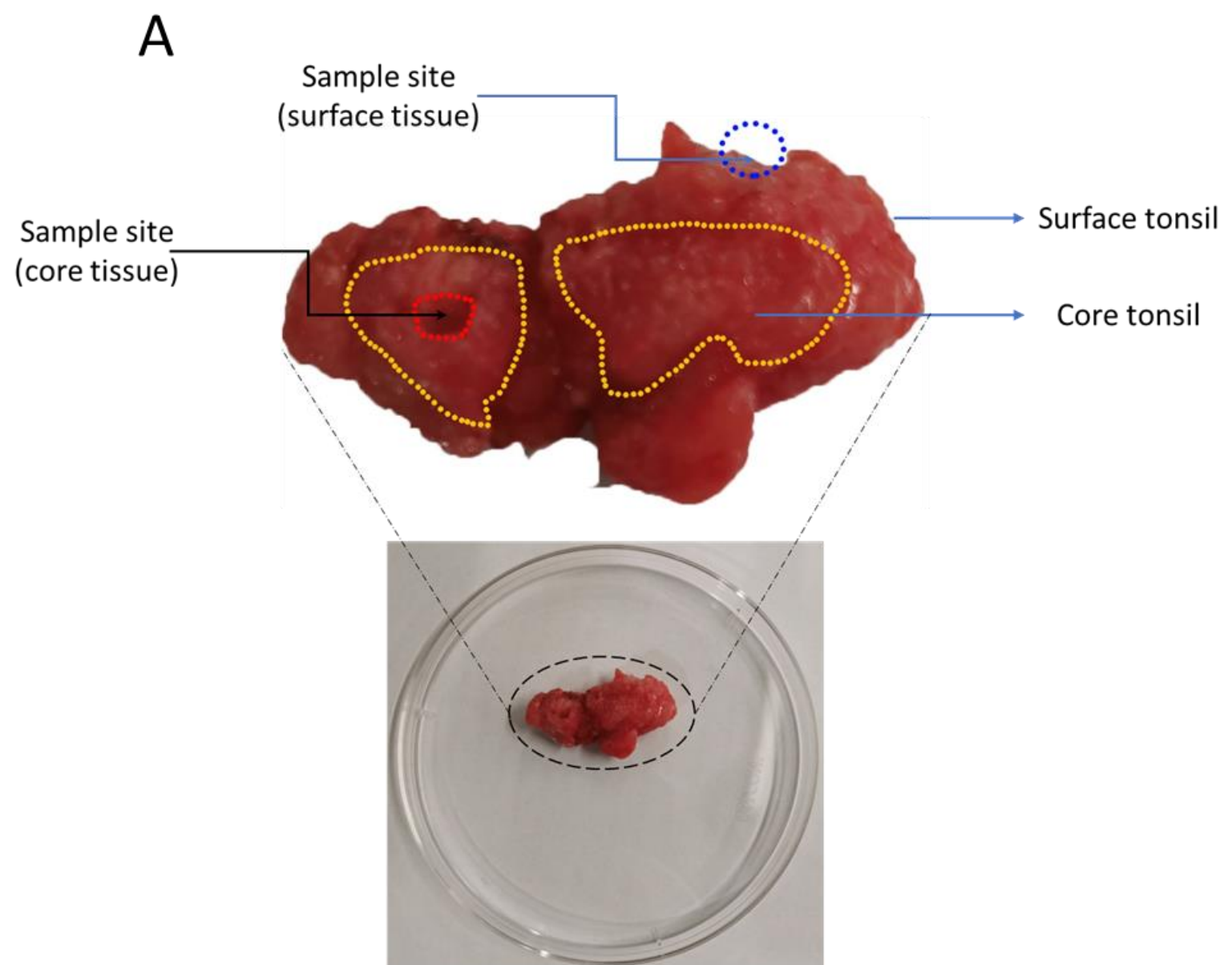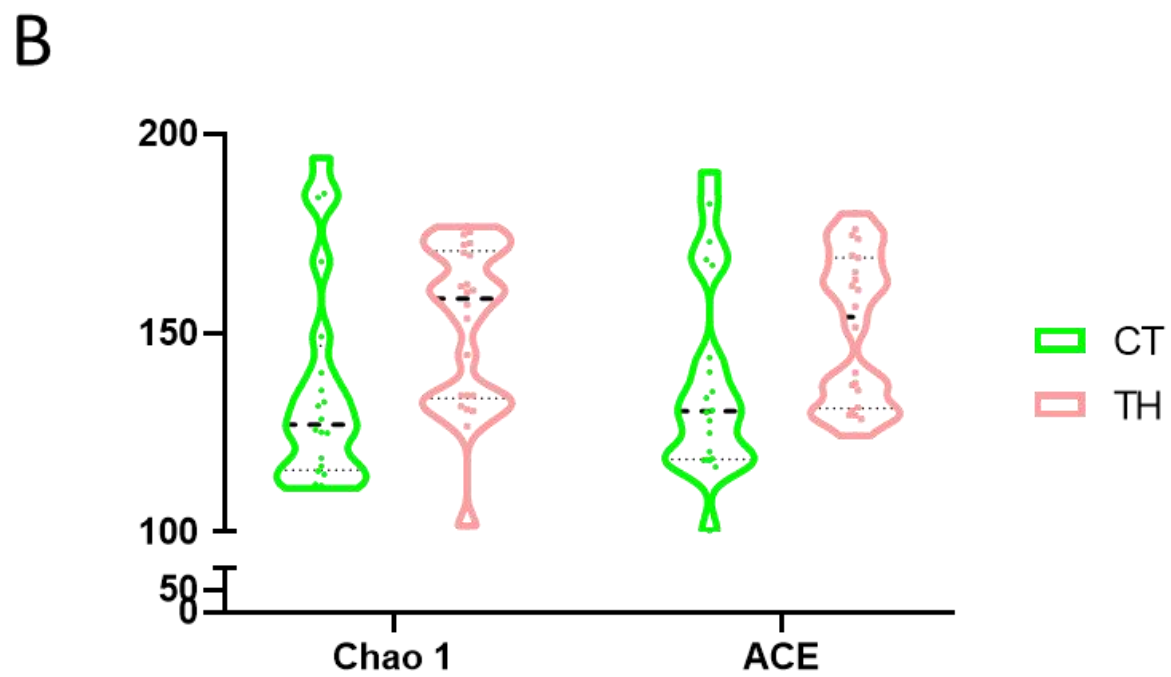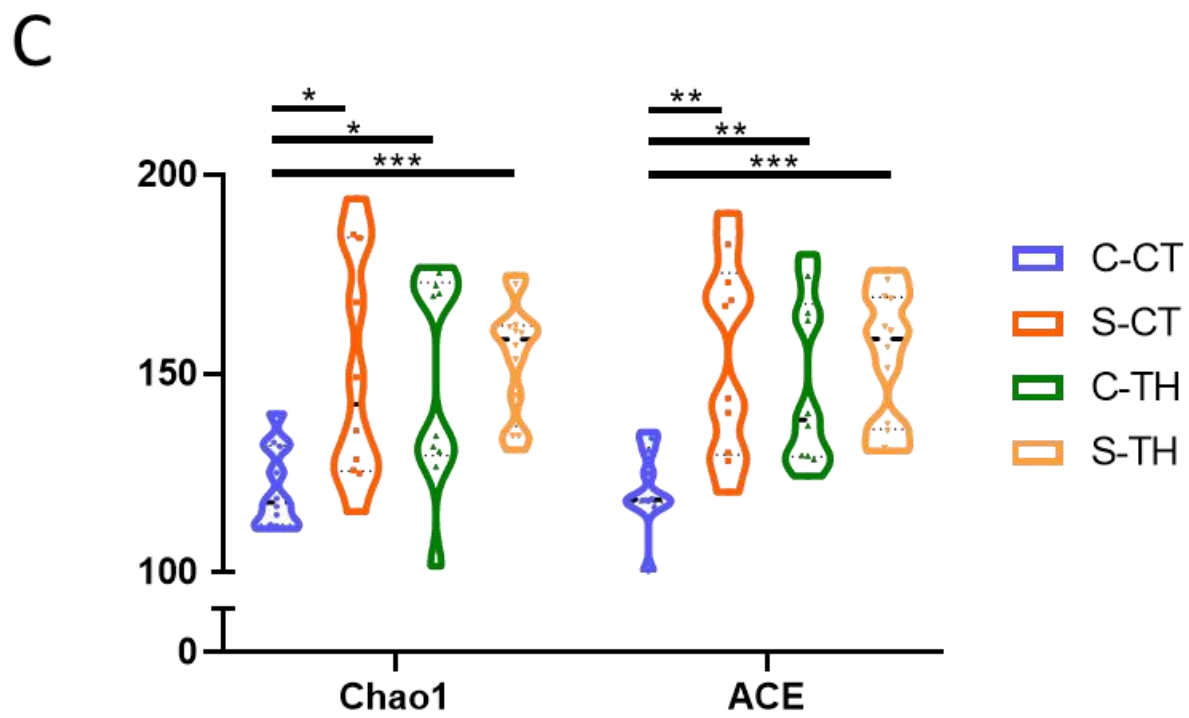

Supplement: FIG S1 [file msystems.01302-20-sf001.pdf]

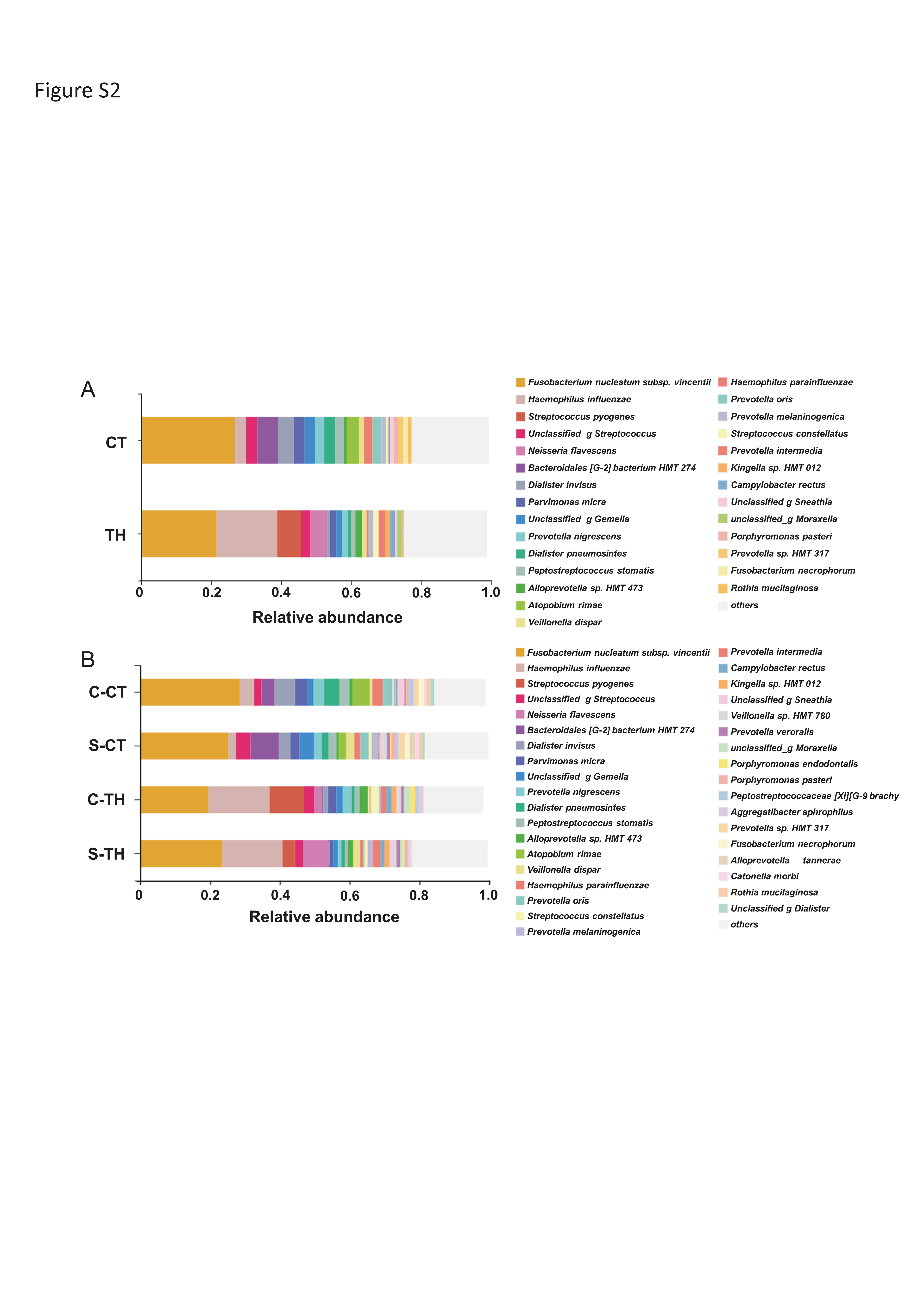

Supplement: FIG S2 [file msystems.01302-20-sf002.tif]

A

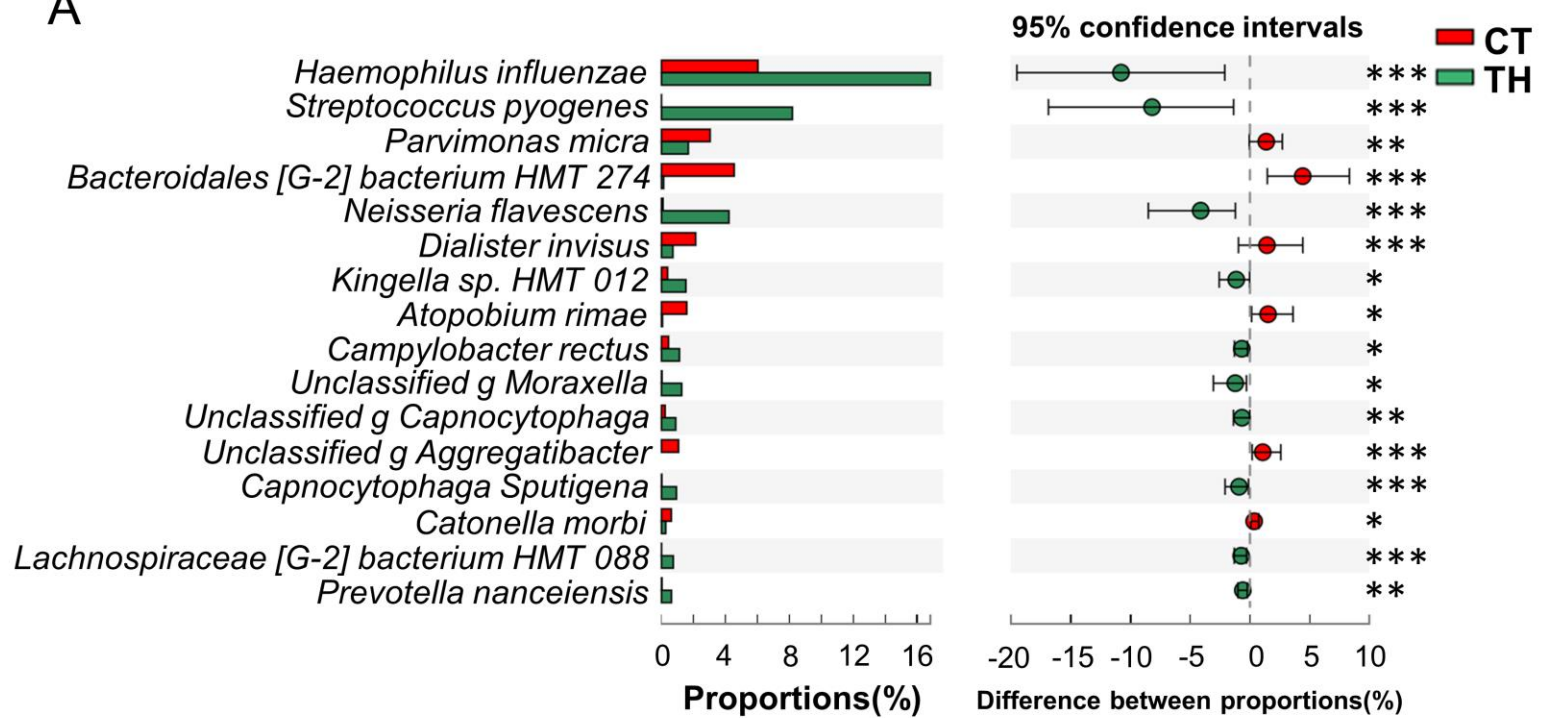

B

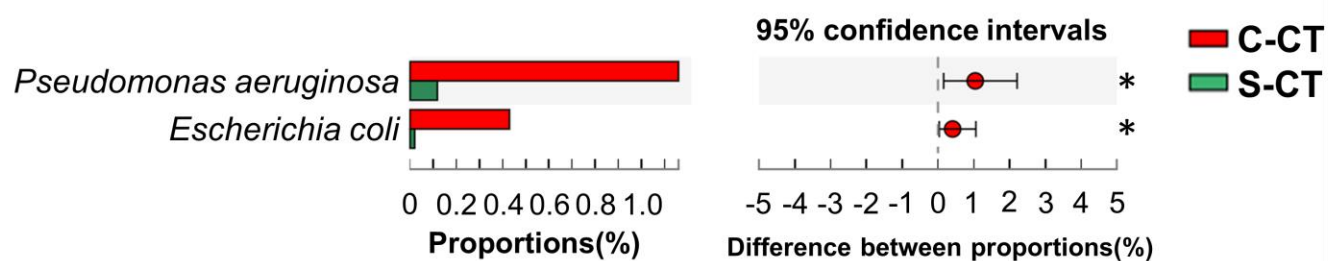

C

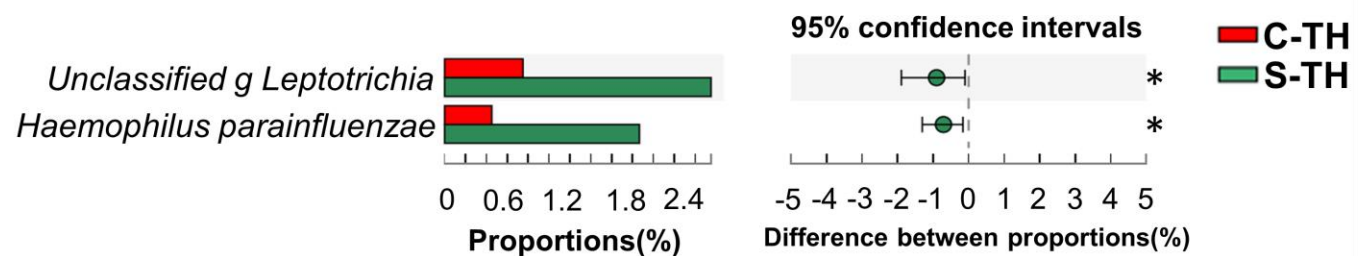

D

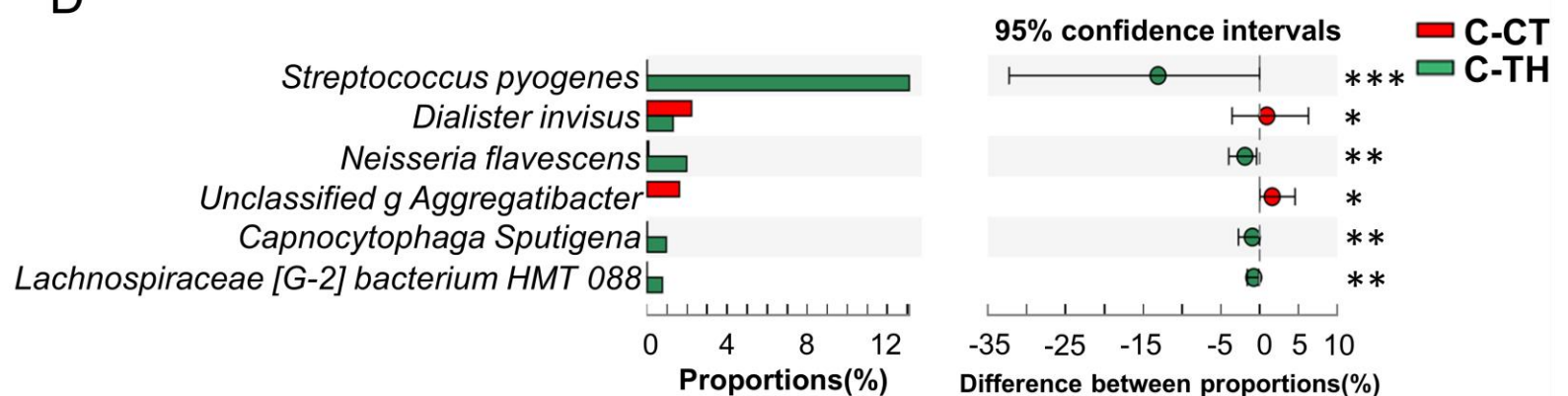

E

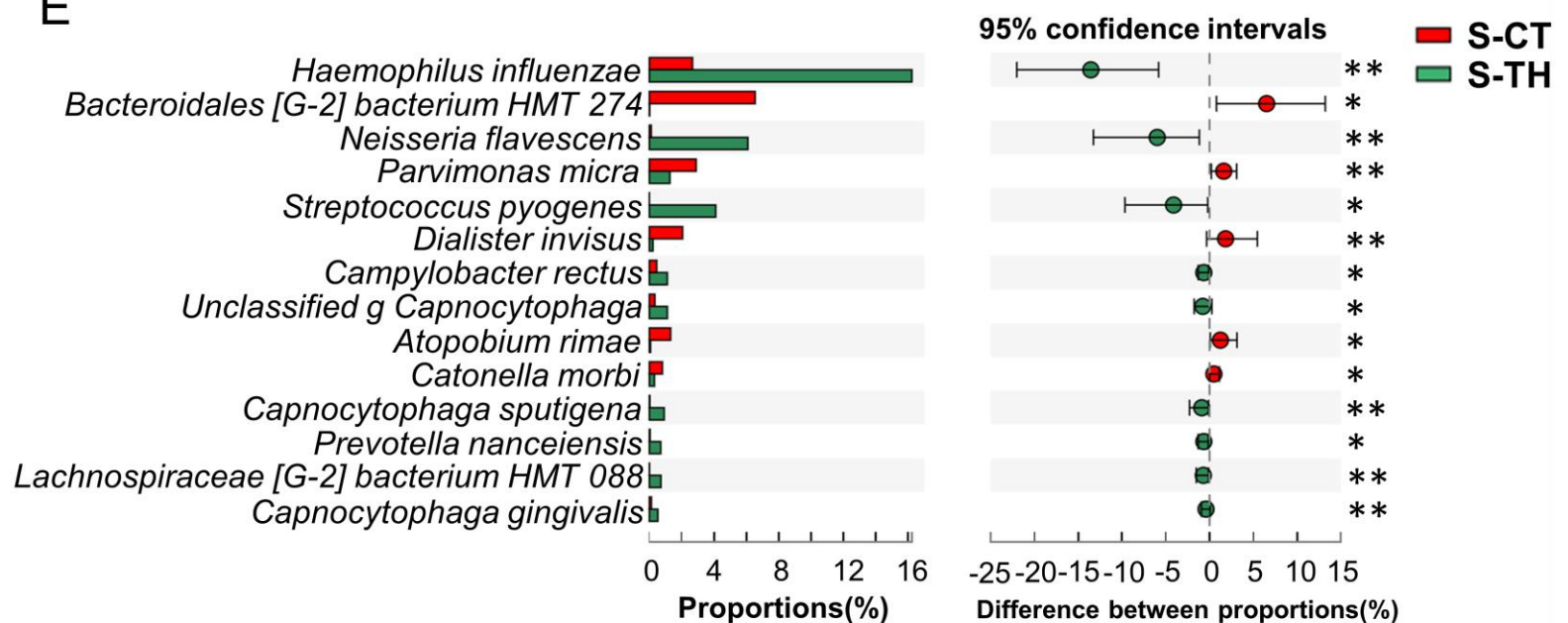

Supplement: FIG S3 [file msystems.01302-20-sf003.pdf]
