# Supplementary material for: Tonsillar Microbiota: a Cross-Sectional Study of Patients with Chronic Tonsillitis or Tonsillar Hypertrophy
Source: mSystems. 2021 Mar 9;6(2):e01302-20. doi: 10.1128/mSystems.01302-20 (PMC8547005; doi:10.1128/mSystems.01302-20)
Supplement: TABLE S1 [file msystems.01302-20-st001.docx]

Table S1. Different microbial composition of core or surface tonsils from chronic tonsillitis (CT) or tonsillar hypertrophy (TH) patients based on a permutational multivariate analysis of variance (PERMANOVA).

| **Compared groups** | **Contribution of each variant** | ***p* value** | |
| --- | --- | --- | --- |
| CT vs. TH | Current group effect | | 0.001 |
|  | Left vs Right tonsil from same patients | | 0.824 |
|  | Gender | | 0.002 |
|  | Age | | 0.002 |
| C-CT vs. S-CT vs. C-TH vs. S-TH | Current group effect | | 0.002 |
|  | Left vs Right tonsil from same patients | | 0.830 |
|  | Gender | | 0.002 |
|  | Age | | 0.002 |
| C-CT vs. S-CT | Current group effect | | 0.322 |
|  | Left vs Right tonsil from same patients | | 0.560 |
|  | Gender | | 0.001 |
|  | Age | | 0.006 |
| C-TH vs. S-TH | Current group effect | | 0.420 |
|  | Left vs Right tonsil from same patients | | 0.728 |
|  | Gender | | 0.006 |
|  | Age | | 0.002 |
| C-CT vs. C-TH | Current group effect | | 0.014 |
|  | Left vs Right tonsil from same patients | | 0.916 |
|  | Gender | | 0.326 |
|  | Age | | 0.096 |
| S-CT vs. S-TH | Current group effect | | 0.003 |
|  | Left vs Right tonsil from same patients | | 0.989 |
|  | Gender | | 0.027 |
|  | Age | | 0.027 |
