# Supplementary material for: Tonsillar Microbiota: a Cross-Sectional Study of Patients with Chronic Tonsillitis or Tonsillar Hypertrophy
Source: mSystems. 2021 Mar 9;6(2):e01302-20. doi: 10.1128/mSystems.01302-20 (PMC8547005; doi:10.1128/mSystems.01302-20)
Supplement: TABLE S2 [file msystems.01302-20-st002.docx]

Table S2. Identification of key genera based on calculated degree centrality, closeness centrality, and betweenness centrality using Python package “NetworkX”.

| **Node Name** | **Degree Centrality** | **Closeness Centrality** | **Betweenness Centrality** |
| --- | --- | --- | --- |
| *Dialister* | 0.2766 | 0.4896 | 0.2176 |
| *Treponema* | 0.2766 | 0.4653 | 0.1018 |
| *Prevotella* | 0.2553 | 0.4700 | 0.1215 |
| *Tannerella* | 0.2128 | 0.4519 | 0.0658 |
| *Catonella* | 0.2128 | 0.4519 | 0.0568 |
| *Parvimonas* | 0.2128 | 0.4159 | 0.0231 |
| *Bergeyella* | 0.2128 | 0.3821 | 0.0399 |
| *Neisseria* | 0.2128 | 0.3821 | 0.0457 |
| *Veillonella* | 0.1915 | 0.3561 | 0.0273 |
| *Bacteroidetes [G-3]* | 0.1702 | 0.4087 | 0.0395 |
